# Supplementary material for: Modeling glioblastoma heterogeneity as a dynamic network of cell states
Source: Mol Syst Biol. 2021 Sep 16;17(9):e10105. doi: 10.15252/msb.202010105 (PMC8444284; doi:10.15252/msb.202010105)
Supplement: Supplementary file 6 — Source Data for Figure 5 [file MSB-17-e10105-s004.zip › Figure5A_sourcedata/GSEA_3017/nosigdb_stateC.GseaPreranked.1622795002785/gsea_report_for_na_neg_1622795002785.html]

Report for na\_neg 1622795002785 [GSEA]

| GS  follow link to MSigDB | GS DETAILS | SIZE | ES | NES | NOM p-val | FDR q-val | FWER p-val | RANK AT MAX | LEADING EDGE || 1 | RICHARDS\_ZHONG\_NATURE\_2018\_ASTROCYTES\_UPREG | Details ... | 74 | -0.62 | -3.98 | 0.000 | 0.000 | 0.000 | 157 | tags=73%, list=26%, signal=87% |
| 2 | RICHARDS\_NOWAKOWSKI\_SCIENCE\_2017\_ASTROCYTE\_UPREG | Details ... | 105 | -0.49 | -3.42 | 0.000 | 0.000 | 0.000 | 162 | tags=59%, list=27%, signal=67% |
| 3 | RICHARDS\_LIDDELOW\_NATURE\_2017\_A2\_REACTIVE\_ASTROCYTES | Details ... | 16 | -0.82 | -3.17 | 0.000 | 0.000 | 0.000 | 60 | tags=81%, list=10%, signal=88% |
| 4 | SUVA\_AC\_PMID\_31327527 | Details ... | 24 | -0.67 | -3.06 | 0.000 | 0.000 | 0.000 | 153 | tags=83%, list=26%, signal=108% |
| 5 | OLSEN\_SCPLIKE | Details ... | 34 | -0.59 | -3.05 | 0.000 | 0.000 | 0.000 | 143 | tags=71%, list=24%, signal=88% |
| 6 | RICHARDS\_NEFTEL\_CELL\_2019\_AC | Details ... | 24 | -0.67 | -3.04 | 0.000 | 0.000 | 0.000 | 153 | tags=83%, list=26%, signal=108% |
| 7 | RICHARDS\_NOWAKOWSKI\_SCIENCE\_2017\_ENDOTHELIAL\_UPREG | Details ... | 133 | -0.41 | -3.01 | 0.000 | 0.000 | 0.000 | 374 | tags=93%, list=63%, signal=194% |
| 8 | RICHARDS\_NOWAKOWSKI\_SCIENCE\_2017\_U1\_UPREG | Details ... | 133 | -0.41 | -2.94 | 0.000 | 0.000 | 0.000 | 267 | tags=71%, list=45%, signal=99% |
| 9 | RICHARDS\_NOWAKOWSKI\_SCIENCE\_2017\_ORG\_UPREG | Details ... | 156 | -0.39 | -2.92 | 0.000 | 0.000 | 0.000 | 222 | tags=59%, list=37%, signal=69% |
| 10 | VANGRONINGEN\_MES | Details ... | 65 | -0.47 | -2.90 | 0.000 | 0.000 | 0.000 | 260 | tags=82%, list=44%, signal=129% |
| 11 | SUVA\_MES1\_PMID\_31327527 | Details ... | 20 | -0.66 | -2.84 | 0.000 | 0.000 | 0.000 | 65 | tags=60%, list=11%, signal=65% |
| 12 | RICHARDS\_NOWAKOWSKI\_SCIENCE\_2017\_MURAL\_UPREG | Details ... | 80 | -0.43 | -2.81 | 0.000 | 0.000 | 0.000 | 259 | tags=74%, list=43%, signal=113% |
| 13 | RICHARDS\_NEFTEL\_CELL\_2019\_MES1 | Details ... | 20 | -0.66 | -2.79 | 0.000 | 0.000 | 0.000 | 65 | tags=60%, list=11%, signal=65% |
| 14 | RICHARDS\_NOWAKOWSKI\_SCIENCE\_2017\_VRG\_UPREG | Details ... | 81 | -0.41 | -2.70 | 0.000 | 0.000 | 0.000 | 186 | tags=59%, list=31%, signal=74% |
| 15 | NB\_KI\_CLUSTER18 | Details ... | 28 | -0.56 | -2.69 | 0.000 | 0.000 | 0.000 | 88 | tags=54%, list=15%, signal=60% |
| 16 | GANDAL\_AUTISM\_UP\_PMID29439242 | Details ... | 24 | -0.59 | -2.69 | 0.000 | 0.000 | 0.000 | 132 | tags=67%, list=22%, signal=82% |
| 17 | IVY\_CTPAN | Details ... | 34 | -0.52 | -2.66 | 0.000 | 0.000 | 0.000 | 100 | tags=53%, list=17%, signal=60% |
| 18 | NB\_KI\_CLUSTER17 | Details ... | 31 | -0.52 | -2.64 | 0.000 | 0.000 | 0.000 | 290 | tags=97%, list=49%, signal=178% |
| 19 | RICHARDS\_NOWAKOWSKI\_SCIENCE\_2017\_TRG\_UPREG | Details ... | 134 | -0.36 | -2.64 | 0.000 | 0.000 | 0.000 | 222 | tags=58%, list=37%, signal=72% |
| 20 | RICHARDS\_CAHOY\_JNEUROSCI\_2008\_ASTROCYTE | Details ... | 143 | -0.35 | -2.62 | 0.000 | 0.000 | 0.000 | 179 | tags=48%, list=30%, signal=52% |
| 21 | RICHARDS\_ZHONG\_NATURE\_2018\_OPC\_UPREG |  | 37 | -0.48 | -2.57 | 0.000 | 0.000 | 0.000 | 229 | tags=78%, list=38%, signal=119% |
| 22 | OLSEN\_MESENCHYMAL |  | 28 | -0.53 | -2.54 | 0.000 | 0.000 | 0.001 | 245 | tags=86%, list=41%, signal=139% |
| 23 | RICHARDS\_NOWAKOWSKI\_SCIENCE\_2017\_MICROGLIA\_UPREG |  | 70 | -0.39 | -2.48 | 0.000 | 0.000 | 0.002 | 361 | tags=93%, list=60%, signal=207% |
| 24 | LARSSON\_STATE\_6 |  | 27 | -0.51 | -2.46 | 0.000 | 0.000 | 0.003 | 174 | tags=67%, list=29%, signal=90% |
| 25 | DOROSZKO\_U3180MG\_CLUSTER\_5 |  | 17 | -0.61 | -2.44 | 0.000 | 0.000 | 0.004 | 183 | tags=82%, list=31%, signal=115% |
| 26 | RICHARDS\_NOWAKOWSKI\_SCIENCE\_2017\_OPC\_UPREG |  | 75 | -0.37 | -2.41 | 0.000 | 0.000 | 0.004 | 163 | tags=51%, list=27%, signal=61% |
| 27 | FELDMAN\_2020\_M-EARLYG1 |  | 35 | -0.46 | -2.41 | 0.000 | 0.000 | 0.004 | 103 | tags=49%, list=17%, signal=55% |
| 28 | GAROFANO\_IAVARONE\_GPM\_SUBTYPE |  | 103 | -0.34 | -2.39 | 0.000 | 0.000 | 0.006 | 315 | tags=77%, list=53%, signal=134% |
| 29 | GANDAL\_SCHIZOPHRENIA\_UP\_PMID29439242 |  | 28 | -0.45 | -2.12 | 0.000 | 0.002 | 0.076 | 162 | tags=61%, list=27%, signal=79% |
| 30 | DOROSZKO\_U3013MG\_CLUSTER\_3 |  | 17 | -0.52 | -2.08 | 0.000 | 0.003 | 0.103 | 127 | tags=59%, list=21%, signal=73% |
| 31 | RICHARDS\_NOWAKOWSKI\_SCIENCE\_2017\_CHOROID\_UPREG |  | 60 | -0.33 | -2.01 | 0.001 | 0.006 | 0.184 | 352 | tags=88%, list=59%, signal=194% |
| 32 | LARSSON\_STATE\_2 |  | 24 | -0.44 | -2.00 | 0.000 | 0.006 | 0.191 | 151 | tags=58%, list=25%, signal=75% |
| 33 | POLLEN\_OUTERRADIALGLIA\_PMID\_26406371 |  | 19 | -0.47 | -1.99 | 0.002 | 0.006 | 0.204 | 147 | tags=63%, list=25%, signal=81% |
| 34 | PARADA\_T1\_UP\_26461091 |  | 18 | -0.49 | -1.99 | 0.005 | 0.006 | 0.205 | 89 | tags=50%, list=15%, signal=57% |
| 35 | GANDAL\_IBD\_UP\_PMID29439242 |  | 16 | -0.50 | -1.98 | 0.012 | 0.007 | 0.224 | 237 | tags=81%, list=40%, signal=131% |
| 36 | RICHARDS\_NOWAKOWSKI\_SCIENCE\_2017\_GLYC\_UPREG |  | 37 | -0.37 | -1.96 | 0.006 | 0.007 | 0.247 | 187 | tags=57%, list=31%, signal=78% |
| 37 | OUTERRADIALGLIA |  | 19 | -0.47 | -1.94 | 0.003 | 0.008 | 0.289 | 147 | tags=63%, list=25%, signal=81% |
| 38 | RICHARDS\_NOWAKOWSKI\_SCIENCE\_2017\_MGE\_RG1\_UPREG |  | 49 | -0.34 | -1.93 | 0.003 | 0.009 | 0.306 | 226 | tags=63%, list=38%, signal=93% |
| 39 | LARSSON\_STATE\_1 |  | 30 | -0.39 | -1.92 | 0.003 | 0.009 | 0.325 | 132 | tags=47%, list=22%, signal=57% |
| 40 | LARSSON\_STATE\_3 |  | 25 | -0.40 | -1.89 | 0.001 | 0.011 | 0.393 | 160 | tags=60%, list=27%, signal=79% |
| 41 | DOROSZKO\_U3054MG\_CLUSTER\_2 |  | 48 | -0.31 | -1.79 | 0.005 | 0.022 | 0.639 | 102 | tags=33%, list=17%, signal=37% |
| 42 | RICHARDS\_ZHONG\_NATURE\_2018\_MICROGLIA\_UPREG |  | 33 | -0.35 | -1.78 | 0.015 | 0.024 | 0.681 | 237 | tags=67%, list=40%, signal=104% |
| 43 | DOROSZKO\_U3054MG\_CLUSTER\_5 |  | 17 | -0.42 | -1.70 | 0.026 | 0.039 | 0.854 | 353 | tags=100%, list=59%, signal=238% |
| 44 | RICHARDS\_CAHOY\_JNEUROSCI\_2016\_CULTURED\_ASTROGLIA |  | 145 | -0.22 | -1.67 | 0.008 | 0.045 | 0.904 | 115 | tags=28%, list=19%, signal=26% |
| 45 | ALMSTEDT\_VGR\_DIFF |  | 104 | -0.24 | -1.67 | 0.013 | 0.044 | 0.908 | 178 | tags=43%, list=30%, signal=51% |
| 46 | RICHARDS\_CAHOY\_JNEUROSCI\_2011\_ASTRO\_MATURE |  | 41 | -0.30 | -1.67 | 0.029 | 0.044 | 0.908 | 114 | tags=34%, list=19%, signal=39% |
| 47 | DOROSZKO\_PERIVASCULAR\_INVASION |  | 17 | -0.41 | -1.66 | 0.028 | 0.044 | 0.913 | 214 | tags=71%, list=36%, signal=107% |
| 48 | NB\_KI\_CLUSTER11 |  | 17 | -0.41 | -1.65 | 0.038 | 0.047 | 0.935 | 256 | tags=76%, list=43%, signal=130% |
| 49 | DOROSZKO\_U3054MG\_CLUSTER\_0 |  | 17 | -0.41 | -1.64 | 0.021 | 0.050 | 0.940 | 239 | tags=71%, list=40%, signal=114% |
| 50 | RICHARDS\_VERHAAK\_CANCERCELL\_2010\_MESENCHYMAL |  | 21 | -0.37 | -1.63 | 0.039 | 0.051 | 0.948 | 361 | tags=95%, list=60%, signal=232% |
| 51 | SINGH\_SOX2\_DOWNREGULATED\_TRANSCRIPTS\_PMID\_28122245 |  | 15 | -0.42 | -1.62 | 0.050 | 0.054 | 0.962 | 203 | tags=67%, list=34%, signal=98% |
| 52 | RICHARDS\_CAHOY\_JNEUROSCI\_2015\_ASTRO\_IN\_VIVO |  | 118 | -0.22 | -1.60 | 0.024 | 0.056 | 0.972 | 276 | tags=59%, list=46%, signal=89% |
| 53 | GANDAL\_BIPOLAR\_UP\_PMID29439242 |  | 17 | -0.40 | -1.60 | 0.034 | 0.057 | 0.975 | 201 | tags=65%, list=34%, signal=95% |
| 54 | GANDAL\_ALCOHOLADDICTION\_DN\_PMID29439242 |  | 15 | -0.42 | -1.58 | 0.053 | 0.062 | 0.982 | 183 | tags=67%, list=31%, signal=94% |
| 55 | PARADA\_T2\_UP\_26461091 |  | 15 | -0.40 | -1.57 | 0.053 | 0.066 | 0.984 | 146 | tags=53%, list=24%, signal=69% |
| 56 | FELDMAN\_2020\_NEURAL G0 |  | 96 | -0.22 | -1.56 | 0.034 | 0.069 | 0.990 | 108 | tags=29%, list=18%, signal=30% |
| 57 | RICHARDS\_VERHAAK\_CANCERCELL\_2010\_CLASSICAL |  | 18 | -0.37 | -1.55 | 0.069 | 0.070 | 0.992 | 196 | tags=61%, list=33%, signal=88% |
| 58 | TISIG\_PMID\_32649888 |  | 18 | -0.38 | -1.53 | 0.064 | 0.078 | 0.992 | 217 | tags=67%, list=36%, signal=102% |
| 59 | FELDMAN\_2020\_LATEG1 |  | 38 | -0.29 | -1.51 | 0.069 | 0.086 | 0.999 | 178 | tags=47%, list=30%, signal=63% |
| 60 | KLING\_GBM\_PHENO\_PMID\_27667176 |  | 24 | -0.32 | -1.44 | 0.099 | 0.119 | 1.000 | 81 | tags=33%, list=14%, signal=37% |
| 61 | DOROSZKO\_U3054MG\_CLUSTER\_10 |  | 29 | -0.29 | -1.43 | 0.109 | 0.120 | 1.000 | 346 | tags=86%, list=58%, signal=195% |
| 62 | LARSSON\_STATE\_5 |  | 20 | -0.32 | -1.36 | 0.152 | 0.168 | 1.000 | 219 | tags=60%, list=37%, signal=92% |
| 63 | DOROSZKO\_U3054MG\_CLUSTER\_3 |  | 24 | -0.30 | -1.35 | 0.153 | 0.172 | 1.000 | 327 | tags=79%, list=55%, signal=168% |
| 64 | DOROSZKO\_U3180MG\_CLUSTER\_2 |  | 54 | -0.22 | -1.30 | 0.153 | 0.213 | 1.000 | 138 | tags=35%, list=23%, signal=42% |
| 65 | NB\_KI\_CLUSTER14 |  | 24 | -0.29 | -1.29 | 0.178 | 0.220 | 1.000 | 371 | tags=92%, list=62%, signal=232% |
| 66 | RICHARDS\_CAHOY\_JNEUROSCI\_2012\_OPC |  | 230 | -0.16 | -1.27 | 0.143 | 0.230 | 1.000 | 197 | tags=38%, list=33%, signal=35% |
| 67 | FELDMAN\_2020\_G2-M |  | 56 | -0.21 | -1.27 | 0.190 | 0.229 | 1.000 | 103 | tags=29%, list=17%, signal=31% |
| 68 | DOROSZKO\_U3054MG\_CLUSTER\_9 |  | 25 | -0.27 | -1.24 | 0.219 | 0.260 | 1.000 | 222 | tags=56%, list=37%, signal=85% |
| 69 | FELDMAN\_2020\_LOWRNA |  | 127 | -0.17 | -1.20 | 0.215 | 0.300 | 1.000 | 114 | tags=25%, list=19%, signal=25% |
| 70 | RICHARDS\_NOWAKOWSKI\_SCIENCE\_2017\_EN\_V1\_3\_UPREG |  | 58 | -0.19 | -1.18 | 0.249 | 0.310 | 1.000 | 190 | tags=41%, list=32%, signal=55% |
| 71 | RICHARDS\_NOWAKOWSKI\_SCIENCE\_2017\_EN\_PFC2\_UPREG |  | 76 | -0.18 | -1.16 | 0.275 | 0.326 | 1.000 | 192 | tags=41%, list=32%, signal=52% |
| 72 | GAROFANO\_IAVARONE\_MTC\_SUBTYPE |  | 20 | -0.27 | -1.14 | 0.299 | 0.344 | 1.000 | 97 | tags=30%, list=16%, signal=35% |
| 73 | RICHARDS\_CAHOY\_JNEUROSCI\_2014\_MOG\_POS |  | 54 | -0.19 | -1.13 | 0.299 | 0.352 | 1.000 | 222 | tags=48%, list=37%, signal=70% |
| 74 | RICHARDS\_NOWAKOWSKI\_SCIENCE\_2017\_EN\_V1\_2\_UPREG |  | 65 | -0.18 | -1.11 | 0.325 | 0.379 | 1.000 | 196 | tags=42%, list=33%, signal=55% |
| 75 | RICHARDS\_NOWAKOWSKI\_SCIENCE\_2017\_U4\_UPREG |  | 92 | -0.16 | -1.04 | 0.423 | 0.459 | 1.000 | 179 | tags=36%, list=30%, signal=43% |
| 76 | RICHARDS\_NOWAKOWSKI\_SCIENCE\_2017\_EN\_PFC3\_UPREG |  | 35 | -0.18 | -0.97 | 0.494 | 0.550 | 1.000 | 196 | tags=43%, list=33%, signal=60% |
| 77 | RICHARDS\_CAHOY\_JNEUROSCI\_2009\_NEURON |  | 70 | -0.14 | -0.91 | 0.587 | 0.637 | 1.000 | 192 | tags=37%, list=32%, signal=48% |
| 78 | RICHARDS\_INHOUSE\_BULKRNASEQ\_2019\_DEVELOPMENTALGSC |  | 130 | -0.12 | -0.89 | 0.638 | 0.648 | 1.000 | 111 | tags=21%, list=19%, signal=20% |
| 79 | RICHARDS\_CAHOY\_JNEUROSCI\_2013\_OL\_MYEL |  | 74 | -0.14 | -0.87 | 0.636 | 0.670 | 1.000 | 222 | tags=42%, list=37%, signal=58% |
| 80 | NB\_KI\_CLUSTER23 |  | 23 | -0.17 | -0.77 | 0.761 | 0.801 | 1.000 | 369 | tags=83%, list=62%, signal=208% |
| 81 | DOROSZKO\_U3054MG\_CLUSTER\_8 |  | 19 | -0.16 | -0.67 | 0.835 | 0.899 | 1.000 | 26 | tags=11%, list=4%, signal=11% |
| 82 | RICHARDS\_NOWAKOWSKI\_SCIENCE\_2017\_NEN\_EARLY1\_UPREG |  | 47 | -0.12 | -0.67 | 0.871 | 0.892 | 1.000 | 192 | tags=36%, list=32%, signal=49% |
| 83 | RICHARDS\_NOWAKOWSKI\_SCIENCE\_2017\_EN\_PFC1\_UPREG |  | 74 | -0.09 | -0.60 | 0.949 | 0.939 | 1.000 | 273 | tags=50%, list=46%, signal=81% |
| 84 | RICHARDS\_CAHOY\_JNEUROSCI\_2008\_OLIGODENDROCYTE |  | 88 | -0.08 | -0.56 | 0.959 | 0.951 | 1.000 | 193 | tags=34%, list=32%, signal=43% |
Table: Gene sets enriched in phenotype **na **[plain text format]****
